# Population-level effects of clinical immunity to malaria

**DOI:** 10.1186/1471-2334-13-428

**Published:** 2013-09-11

**Authors:** Lindsay T Keegan, Jonathan Dushoff

**Affiliations:** 1 Department of Biology, McMaster University, Hamilton, Ontario, Canada

## Abstract

**Background:**

Despite a resurgence in control efforts, malaria remains a serious public-health problem, causing millions of deaths each year. One factor that complicates malaria-control efforts is clinical immunity, the acquired immune response that protects individuals from symptoms despite the presence of parasites. Clinical immunity protects individuals against disease, but its effects at the population level are complex. It has been previously suggested that under certain circumstances, malaria is *bistable*: it can persist, if established, in areas where it would not be able to invade. This phenomenon has important implications for control: in areas where malaria is bistable, if malaria could be eliminated until immunity wanes, it would not be able to re-invade.

**Methods:**

Here, we formulate an analytically tractable, dynamical model of malaria transmission to explore the possibility that clinical immunity can lead to bistable malaria dynamics. We summarize what is known and unknown about the parameters underlying this simple model, and solve the model to find a criterion that determines under which conditions we expect bistability to occur.

**Results:**

We show that bistability can only occur when clinically immune individuals are more “effective” at transmitting malaria than naïve individuals are. We show how this “effectiveness” includes susceptibility, ability to transmit, and duration of infectiousness. We also show that the amount of extra effectiveness necessary depends on the ratio between the duration of infectiousness and the time scale at which immunity is lost. Thus, if the duration of immunity is long, even a small amount of extra transmission effectiveness by clinically immune individuals could lead to bistability.

**Conclusions:**

We demonstrate a simple, plausible mechanism by which clinical immunity may be causing bistability in human malaria transmission. We suggest that simple summary parameters – in particular, the relative transmission effectiveness of clinically immune individuals and the time scale at which clinical immunity is lost – are key to determining where and whether bistability is happening. We hope these findings will guide future efforts to measure transmission parameters and to guide malaria control efforts.

## Background

Despite extensive efforts to eradicate it, malaria caused by *Plasmodium falciparum* remains a significant problem resulting in millions of cases and 660,000 deaths in 2010 [1]. A characteristic of *falciparum* malaria disease that complicates control efforts is clinical immunity – an immune response that develops with exposure to parasites and provides protection against the clinical symptoms of malaria, despite the presence of parasites [2]. Although clinical immunity protects individuals against disease, its effects at the population level are complex.

Malaria is highly variable from region to region, further complicating analysis. Geographic variation in average disease burden (endemicity) leads to variation in acquired immunity [3]. Malaria endemicity ranges from “holoendemic” (defined as having a parasite ratio (PR, the percentage of subjects with parasites found in the blood) consistently greater than 75% of infants [4]) through “hyperendemic” and “mesoendemic” to “hypoendemic”, defined as having a PR of less than 10% of children age 2–9 [4]. This results in variation in the acquisition of clinical immunity. This variation in endemicity and clinical immunity complicates malaria epidemiology and control.

Clinical immunity to malaria develops after exposure to parasites and varies as endemicity varies [5,6]. In holoendemic regions, exposure to parasitemia is high enough that clinical immunity develops rapidly, and most adults and older children are clinically immune, whereas in hypoendemic regions, most people are not re-infected often enough to develop clinical immunity [6]. Even after it develops, clinical immunity can be lost in 3-5 years without re-exposure [2,3,5-7]. When individuals first develop clinical immunity, they are only immune to severe symptoms. If re-exposure continues, however, clinical immunity can result in asymptomatic or nearly asymptomatic disease. Full clinical immunity develops slowly and tends to correlate with the onset of puberty [6,8].

An important aspect of clinical immunity is the possibility that clinically immune individuals are particularly effective at transmitting malaria over the duration of their infections. This phenomenon could arise if clinically immune individuals are more infectious to mosquitoes per unit time, or if they stay infectious for longer (perhaps because they are less likely to seek medical treatment), or both. If clinically immune individuals have a higher reproductive rate, this has potential implications at the population level – in some cases, malaria may be spread more effectively in areas where it is already present, all else being equal.

As transmission of malaria decreases, the proportion of the population protected by clinical immunity decreases as well, since clinical immunity is lost. As a result, decreases in transmission can, under some circumstances, lead to an *increase* in morbidity and mortality, because fewer people are protected against the symptoms of malaria [9-11].

Águas et al. [12] have shown that under certain circumstances when clinically immune individuals are more infectious over the duration of their infection, than naïve cases, malaria can persist, if established, in areas where it would not be able to invade. In other words, for some sets of parameters, both an endemic equilibrium and a disease-free equilibrium are stable – a phenomenon known as bistability. Bistability would have important implications for malaria control: in particular, it would imply that there are some areas where, if malaria could be eliminated until clinical immunity wanes, it would not be able to re-invade. Here we use a model of malaria transmission to explore under which conditions we would expect bistability to occur, indicating possible opportunities for malaria elimination.

Malaria elimination has been surprisingly effective in many countries: 75 of the 79 countries that successfully eliminated malaria between 1945 and 2010 remain malaria free, even though many have not sustained control efforts [13]. A recent paper by Chiyaka et al. [14] presents six hypotheses for this phenomenon, and argues that $R_{0}$ may be reduced either by external factors, like demographic and hydrological changes, or by factors driven by malaria elimination itself, for example economic development catalyzed by reduced disease burden, or bistability due to treatment seeking. They argue that, to the extent that malaria elimination reduces $R_{0}$, incentives to aggressively pursue control are increased, since on-going active control efforts will not be required once malaria is eliminated.

In this paper, we first review what is known about infectiousness and susceptibility to infection of clinically immune individuals. We then build a simple transmission model designed to elucidate what factors make bistability likely, and what measurements could shed light on when and whether bistability is likely to be an important phenomenon in malaria dynamics and control. We derive a simple mathematical criterion for how “effective” transmission by clinically immune individuals must be for bistability to occur.

### Effects of clinical immunity on disease transmission

The overall infectiousness of an infected individual is the product of duration of infection, and mean infectiousness. Below, we review what is known about the effects of clinical immunity on these components.

The duration of a malaria infection is highly variable, and treatment-seeking behavior is an important determinant. In a review of population-level studies done on malaria treatment-seeking behavior, McCombie [15] found that treatment rates were correlated with severity of symptoms, and that in Africa, 64–95% of individuals who sought treatment received at least one form of treatment; with the majority of studies reviewed reporting over 90% treatment rate.

It is well known that many clinically immune infections are not even recognized by the individual as malaria. Individuals in hyper- and holo-endemic areas who do not think they have malaria, have been found to test positive at high rates, for example in Ghana [15,16], Senegal [17], and Kenya [18]. Thus, it seems reasonable to suppose that most clinically immune infections are untreated, and last longer than treated clinical infections because of treatment-seeking behavior. Various studies have been done to estimate the duration of untreated malaria infection. Earle et al. [19] observed the duration of infection in children age 5-15 years old and found all of them had cleared the infection within a year. However, most of what is known about duration of infection comes from malariatherapy data. These studies infected malaria-naïve syphilis patients with *P. falciparum* strains with low clinical virulence and found the mean duration of infection to be 200 days [20]. A recent study in a highly endemic region found a similar mean duration of infection to that of the malariatherapy data, however, they found a larger variance in duration of infection with many more infections with shorter duration than found in the malariatherapy data [21]. And in an extreme case, an infection was found 8 years after last known exposure to parasites [22]. However, it’s not clear how the duration of untreated clinically immune infections compares to untreated symptomatic cases. Bruce et al. [23] found that “episodes” of parasitemia lasted longer in children than in adults and suggest that this may be due to clinical immunity [23]. Unfortunately, it is not straightforward to relate their measured episodes to infection clearance (partly because infected individuals may be “super-infected” by other strains).

While clinically immune cases are frequently asymptomatic or of low clinical virulence, data from malariatherapy studies suggest naïve cases may also have a wide range of clinical virulence ranging from high virulence to asymptomatic [24]. Other studies suggest that asymptomatic malaria is not limited to areas of high transmission where exposure-related immunity is expected to develop [25-27]. Thus, not all naïve cases may be terminated with treatment.

Another key component to the population-level effects of clinical immunity is the infectiousness per transmission event of infected individuals. Although malaria transmission is much studied, it remains unclear how parasitemia, gametocytemia, and other factors interact to affect malaria transmission. Gametocyte quantity alone is not sufficient to ensure successful transmission; mosquito uptake of gametocytes depends on a wide variety of factors, including transmission-blocking immunity (TBI) [28-30] and cytokine tumor necrosis factor (TNF) [31,32].

Transmission-blocking immunity (TBI) is a human immune reaction to sexual stages of malaria. TBI develops with exposure to gametocytes and, through a variety of mechanisms, reduces successful transmission of new infections. TBI increases with gametocyte density; consequently, TBI tends to be negatively correlated with clinical immunity [30], but the importance of TBI to population-level transmission is not clear. In one study, transmission-blocking immunity was found to reduce transmission by up to 90%, with higher immunity in the younger age groups [30]. Two other studies, which did not include the youngest age group, failed to find correlations between age and TBI [33,34]; the latter of these found that only 15% of urban and 29% of rural gametocyte carriers had reduced transmission. A model of human infectiousness to mosquitoes found that patterns of EIR across Africa and Papua New Guinea could be explained without invoking TBI [35].

Cytokine TNF is another factor that affects gametocyte success. It is present in the blood serum taken during the crisis of a malaria infection [31,32]. Cytokine TNF is responsible for the loss of infectiousness during peak parasitemia by killing the gametocytes. Gametocytes present during malaria crisis were found dead before entering the mosquito; as well, gametocytes from crisis serum failed to infect mosquitoes even when washed and re-suspended in normal serum [31].

Although there is immunity against gametocytes at peak parasitemia in non-immune individuals, clinically immune infections often have lower gametocytemia as a result of having anti-parasite immunity, conferring protection against high-density parasitemia [7]. There is also evidence that clinically immune individuals are less infectious per bite, but because clinically immune individuals are less likely to seek treatment, they are consistently infectious at low levels for long periods of time and therefore result in producing a large number of new infections over the course of a single clinically immune infectious period [36,37].

As we will see below, susceptibility to infection in clinically immune individuals is also important to the population-level dynamics of malaria. Although a great deal is known about susceptibility to clinical illness, parasitemia or gametocytemia [38,39], much less is known about susceptibility to new infection. Individual susceptibility to new infections is complex, and known to be influenced by genotype, parasite virulence, and specific immunity [38], but there is evidence to believe that clinically immune individuals are about as susceptible to disease as non-immune individuals [40].

### Population-level effects

We investigate the factors underlying bistability with a simple transmission model that accounts for clinical immunity (see Methods). We assume that individuals infected when they are naïve have a probability of becoming clinically immune when they recover from infection, and that clinically immune susceptible individuals lose immunity at some rate if not infected again, meaning that clinical immunity will be maintained when the force of infection is high, and will often wane if the force of infection is low.

To explore the effects that clinically immune individuals have on the population-level disease dynamics, we compare the life-cycle transmission effectiveness of naïve and clinically immune individuals, using subgroup reproductive numbers. These are defined as the average number of secondary infections from a single infectious individual in an otherwise totally susceptible population. We define the reproductive number for naïve cases, $R_{\text{NN}}$, as the average number of secondary infections generated by a single naïvely infectious individual in an otherwise totally naïvely susceptible population. We define the reproductive number for clinically immune cases, $R_{\text{CC}}$, as the average number of secondary infections given by a single clinically immune infectious individual in an otherwise totally clinically immune population.

Because we assume that all individuals are naïve in the absence of infection, the basic reproductive number of our system $R_{0}=R_{\text{NN}}$. We stress that this is the basic reproductive number in the presence of baseline control efforts – in particular, we assume that treatment is always available to those who seek it. This $R_{0}$ will typically differ from the $R_{0}$ that would be calculated in the absence of control [14].

The reproductive numbers determine malaria disease dynamics. When $R_{\text{NN}}>1$ the disease will always persist. When $R_{\text{NN}}\leq 1$ the disease cannot invade. However, there is evidence that under certain circumstances when $R_{\text{CC}}>1>R_{\text{NN}}$, clinically immune individuals can act as a reservoir and allow malaria to remain endemic, even though $R_{\text{NN}}$ drops below one. In this case, both the disease-free equilibrium and an endemic equilibrium are stable, this is an example of “bistability”.

Bistability is typically associated with “backwards bifurcations”. In general, as a disease invades, it reduces its reproductive number R, primarily by reducing the number of susceptibles in the population. In such “forward bifurcations”, we expect the disease to go extinct from any starting conditions when $R_{0}\leq 1$, and to reach a small equilibrium, when $R_{0}$ is just above 1 [41]. When a disease *increases* its reproductive number as it invades, backwards bifurcations occur. In a backwards bifurcation, the disease invades to a non-zero level even when $R_{0}=1$, and will be able to persist above a certain threshold when $R_{0}$ is just below 1 [41].

## Methods

**Figure 1:**
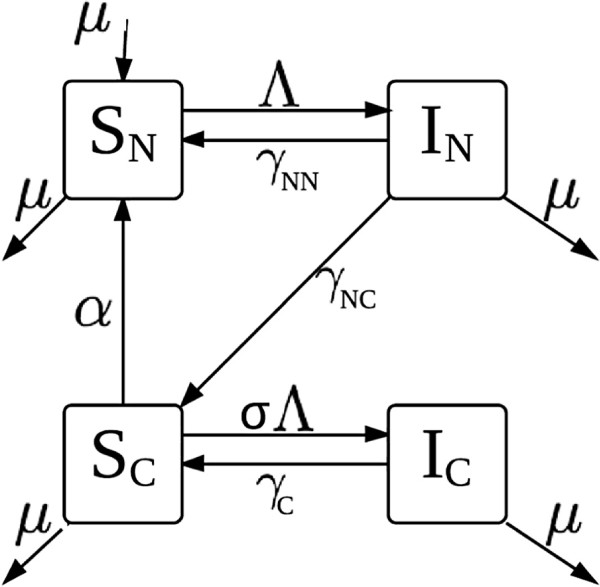
**Compartmental diagram of our malaria transmission model.** Each compartment in the diagram represents a different epidemiological class. Individuals begin in the susceptible naïve class (*S*_*N*_), return there after losing clinical immunity, at a rate *α*, and are born into this class, at a rate *μ*. Individuals in *S*_*N*_ who get infected move to the infected naïve class (*I*_*N*_), at a rate *Λ*. From *I*_*N*_, individuals recover from illness to either the susceptible naïve class (*S*_*N*_), at a rate *γ*_*NN*_, if immunity was not conferred, or to the susceptible clinically immune class (*S*_*C*_), at a rate *γ*_*NC*_ if clinical immunity developed. Individuals who are susceptible clinically immune can either lose immunity at a rate *α* and return to the susceptible naïve class, or they can get infected, at a rate *σΛ*, and become infected clinically immune (*I*_*C*_). Infected clinically immune individuals recover to the susceptible clinically immune class, at a rate *γ*_*C*_. Individuals can die from any of the epidemiological classes and do so at a rate *μ* independent of the level of malaria in the population. Individuals are born and die at the same rate, thus keeping the population size constant.

To explore the dynamics of malaria and determine the conditions in which bistability can occur, we evaluated the following simple transmission model (Figure 1):

(1a) $$\begin{array}{ll} \frac{{\text{dS}}_{N}}{\text{dt}} & =-\Lambda S_{N}+\alpha S_{C}-\mu S_{N}+\mu T+\gamma_{\text{NN}}I_{N}\; \end{array}$$

(1b) $$\begin{array}{ll} \frac{{\text{dI}}_{N}}{\text{dt}} & =\Lambda S_{N}-(\gamma_{\text{NN}}+\gamma_{\text{NC}})I_{N}-\mu I_{N}\; \end{array}$$

(1c) $$\begin{array}{ll} \frac{{\text{dS}}_{C}}{\text{dt}} & =-\sigma \Lambda S_{C}-\alpha S_{C}+\gamma_{\text{NC}}I_{N}+{\gamma {}_{C}I}_{C}-\mu S_{C}\; \end{array}$$

(1d) $$\begin{array}{ll} \frac{{\text{dI}}_{C}}{\text{dt}} & =\sigma \Lambda S_{C}-{\gamma {}_{C}I}_{C}-\mu I_{C}\; \end{array}$$

*S*_*N*_ represents naïve susceptible individuals: individuals who have never been infected with malaria, those who have been infected but have not developed clinical immunity, or who have lost all immunity. When infected with malaria, they move to the clinically infected class (*I*_*N*_). Recovered individuals become immediately susceptible again, but with immunity to clinical symptoms (*S*_*C*_). When non-naïve susceptibles get infected, they acquire clinically immune infections (*I*_*C*_). Each class represents a portion of the population. *τ*_*N*_ and *τ*_*C*_ are the transmission rates of naïve and clinically immune cases, respectively. *γ*_*NN*_ is the rate at which naïve individuals recover without clinical immunity, *γ*_*NC*_ is the rate they recover with clinical immunity, and *γ*_*C*_ is the recovery rate for clinically immune individuals. *α* is the rate at which clinical immunity is lost *σ* is the relative susceptibility to new infection of clinically immune individuals. $\Lambda =\frac{\tau_{N}I_{N}+\tau_{C}I_{C}}{T}$ is the force of infection. Both types of susceptibles (naïve or clinically immune) can be infected by either type of infectious individual (naïve or clinically immune). The total population size is *T*=*S*_*N*_+*I*_*N*_+*S*_*C*_+*I*_*C*_. For this model, $R_{0}=R_{\text{NN}}=\frac{\tau_{N}}{\gamma_{\text{NN}}+\gamma_{\text{NC}}+\mu }$, and $R_{\text{CC}}=\frac{\sigma \tau_{C}}{\gamma_{C}+\mu }$. We define *ρ* to be the ratio of the clinically immune reproductive number to the naïve reproductive number ($R_{\text{CC}}/R_{\text{NN}}$). And $\pi =\frac{\gamma_{\text{NC}}}{\gamma_{\text{NN}}+\gamma_{\text{NC}}}$ is the proportion of naïve infections that recover to become clinically immune.

We used the statistical package R [42] to simulate our model. We held the average durations of infectiousness for naïve and immune individuals (1/*γ*_*NN*_ and 1/*γ*_*C*_) constant at 50 and 200 days respectively. We chose $\pi =\frac{\gamma_{\text{NC}}}{\gamma_{\text{NC}}+\gamma_{\text{NN}}}$ to be 0.5, assuming an equal chance of getting clinical immunity and remaining non-immune after each naïve infection; results with *π*=1 were similar. Transmission coefficients *τ*_*N*_ and *τ*_*C*_ were calculated from $R_{\text{NN}}$ and $R_{\text{CN}}$ which varied as described in the Results section. We chose duration of immunity 1/*α* to be 1282 days (about 3.5 years), and lifespan 1/*μ* to be 25,550 days (about 70 years). The relative susceptibility of clinically immune individuals was chosen to be *σ*=0.7 since clinically immune individuals are about as susceptible as naïve cases; we also tested other values.

R code and Maxima code to replicate all of our results will be made available upon publication.

In building a simple transmission model, we have lumped a variety of biological mechanisms into the parameters. For example, transmission parameters *τ*_*C*_ and *τ*_*N*_ include transmission blocking immunity and the reduction of parasites in clinically immune individuals. The duration of infection parameters *γ*_*NN*_, *γ*_*NC*_, and *γ*_*C*_ take into account treatment seeking behavior (naïve individuals are likely to seek treatment quickly whereas clinically immune individuals are less likely to seek treatment or they wait longer to seek treatment).

## Results

**Figure 2:**
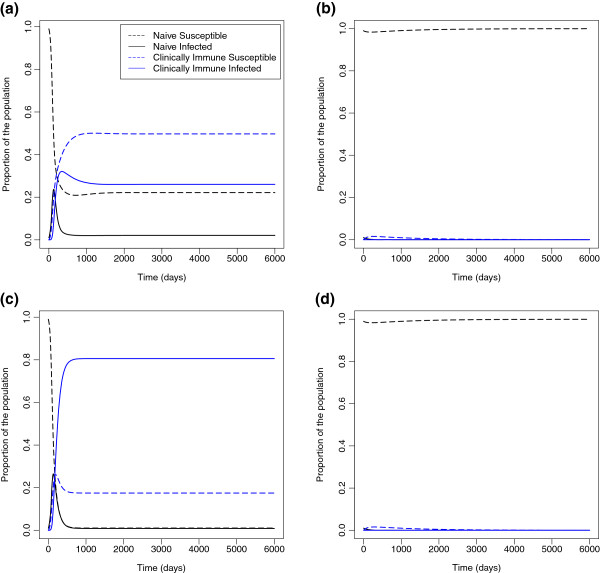
**Simulation of malaria transmission in a naïve population.** We simulated malaria transmission in a population with 95% naïve susceptible and 5% naïve infectious individuals (*S*_*N*_=0.95,*I*_*N*_=0.05,*S*_*C*_=0,*I*_*C*_=0,*N*=1, *α*=0.001, *γ*_*NN*_=0.02, *γ*_*NC*_=0.02, *γ*_*C*_=0.005, *μ*=0.000039, and *σ*=0.7) under two assumptions of *ρ*. Panels a and b show *ρ*=0.5 and panels c and d show *ρ*=3.5**(a)**$R_{\text{NN}}=2>1>R_{\text{CC}}=1$. **(b)**$1>R_{\text{NN}}=0.75>R_{\text{CC}}>0.375$. **(c)**$R_{\text{CC}}=7>R_{\text{NN}}=2>1$. **(d)**$R_{\text{CC}}=2.625>1>R_{\text{NN}}=0.75$.

We simulated our malaria transmission model under two scenarios of infectiousness: overall infectiousness of clinically immune cases was either low ($\rho \equiv R_{\text{CC}}/R_{\text{NN}}=0.8$, panels 2a and 2b) or high (*ρ*=4, panels 2c and 2d). For each scenario, we simulated two different values of $R_{0}\equiv R_{\text{NN}}$. Disease-invasion results are shown in Figure 2. When we start near the disease-free equilibrium, the qualitative behavior is determined by $R_{0}$: when $R_{0}>1$ (panels 2a and 2c), the disease invades and reaches an endemic equilibrium; when $R_{0}<1$, (panels 2b and 2d), the disease does not invade.

**Figure 3:**
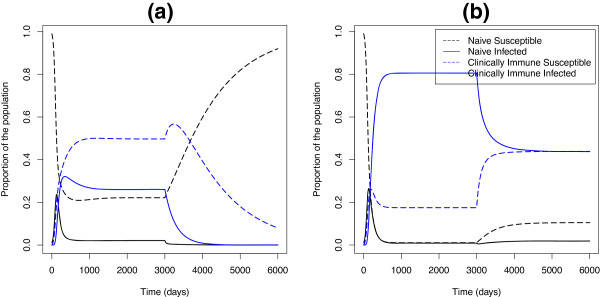
**Simulation of malaria transmission model with control.** Malaria transmission with changing parameters. Here we simulated malaria using the parameters from the first column of Figure 2 until they reached equilibrium at which point we changed the parameters to those in the second column and allowed malaria to reach equilibrium again. **(a)** We used the parameters from Figure 2a from 0 to 3000 and the parameters from Figure 2b from 3000 to 6000. **(b)** We used the parameters from Figure 2c from 0 to 3000 and from Figure 2d from 3000 to 6000.

In the case where underlying parameters can change over time, however, there are striking differences between the scenarios with low and high relative transmission from immune individuals. Figure 3 shows what would happen in a population with endemic malaria if control efforts moved transmission from the first column of Figure 2 to those shown in the second column. Panel 3a behaves as we would expect: when we change the parameters at day 3000, the system moves to the disease-free equilibrium. Panel 3b, however, exhibits bistability. Although the parameters in the latter part of the simulation are consistent with disease extinction, the disease does not go extinct from the equilibrium reached under high transmission, but instead finds a lower endemic equilibrium. Whether or not malaria will remain endemic or die out under parameters consistent with disease extinction is dependent both on $R_{0}$ and on initial conditions.

**Figure 4:**
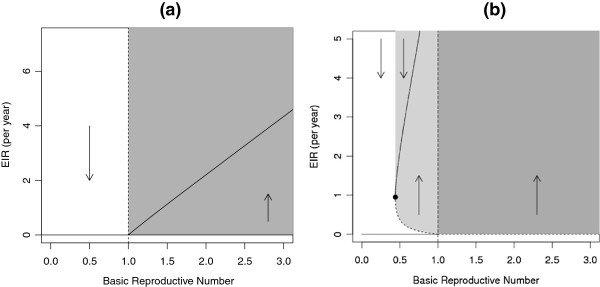
**Bifurcation diagram for our model.** Bifurcation diagram for malaria. The dashed line shows $R_{0}=1$ and the arrows show the behavior of the system: in the white area, to the left of the manifold, the disease will die out while to the right of the manifold, in the shaded area, the disease will persist. The dark-shaded region represents the area in which malaria can invade and persist and the light-shaded area in which it persists, even though it would not invade. In these figures, all parameters are held constant except *τ*_*N*_ and *τ*_*C*_. **(a)** A forward bifurcation occurs at $R_{0}=1$. *ρ*=0.8. **(b)** A backwards bifurcation occurs at $R_{0}=1$. *ρ*=4.

Figure 4 gives a broader perspective on the two scenarios, using “bifurcation diagrams” showing how equilibrium incidence changes as $R_{0}$ increases, while holding the relative infectiousness of clinically immune individuals ($\rho \equiv R_{\text{CC}}/R_{\text{NN}}$) constant. Panel 4a illustrates the scenario where clinically immune individuals are relatively less effective at transmitting disease (*ρ*<1). Here we see the simple, common, relationship between $R_{0}$ and equilibrium incidence. As $R_{0}$ increases past 1, the system moves smoothly from having a globally stable equilibrium at 0, to having a globally stable endemic equilibrium.

Panel 4b shows shows the scenario where clinically immune individuals are relatively more effective at transmitting disease (*ρ*>1). In this case, we see a more complicated pattern, where both $R_{0}$ and initial prevalence affect the final outcome. In particular, if we increase $R_{0}$ smoothly past 1 in a population where the disease is absent, the equilibrium jumps abruptly; if the disease invades, and $R_{0}$ is decreased back below 1 the disease does not necessarily go extinct. Similarly, if $R_{\text{crit}}<R_{0}<1$ (the light gray region of the plot), a temporary intervention that sharply reduces disease prevalence could succeed in eliminating the disease even without a long-term reduction in $R_{0}$.

To be specific, we don’t expect the disease to go extinct once established unless $R_{0}$ is reduced beneath the minimum value for which the endemic equilibrium exists. We call this value $R_{\text{crit}}$ and the force of infection *Λ* that corresponds to it *Λ*_*crit*_. On the forward bifurcation diagram (Figure 4a) $R_{\text{crit}}=1$ and on the backwards bifurcation diagram (Figure 4b) $R_{\text{crit}}$ is precisely the point where the stability of the endemic equilibrium (in a region of bistability) changes from unstable to stable (the black dot on Figure 4b).

In Additional file 1, we show that a backwards bifurcation will occur when the ratio $\rho^{*}=R_{\text{CC}}/R_{\text{NN}}>\rho$, where:

(2) $$\rho^{*}=1+\frac{D}{\pi L}$$

Here *L*=1/(*α*+*μ*) is the duration of immunity, *D*=1/(*γ*_*NC*_+*γ*_*NN*_) is the duration of naïve infection, and $\pi =\frac{\gamma_{\text{NC}}}{\gamma_{\text{NC}}+\gamma_{\text{NN}}}$ is the proportion of people who become clinically immune after a naïve infection. This criterion determines for what parameters bistability can occur when $R_{0}<1$. The value of *ρ*^∗^ is always strictly greater than 1, meaning that bistability only occurs when $R_{\text{CC}}$ exceeds $R_{\text{NN}}$ by a sufficient amount. The amount of excess necessary is determined by how quickly immunity is lost (through death or waning) compared to the duration of infectiousness of the disease: when immunity lasts longer, bistability is more likely.

When *ρ*>*ρ*∗, a backwards bifurcation occurs, resulting in a region of bistability where there exists a stable disease-free equilibrium and a stable endemic equilibrium for the same parameter values. Figure 4b illustrates the backwards bifurcation and the region of bistability (light gray shaded region). Within the region of bistability, if malaria were to be eliminated, it would not be able to re-invade unless $R_{0}$ were increased from below one back above one, making malaria elimination from these regions more sustainable. In order to eliminate malaria from a region of bistability, either the force of infection must be reduced below the unstable endemic equilibrium (dashed curve), or the reproductive number must be reduced below the critical value ($R_{\text{crit}}$), or a combination of these. In Figure 4b, this is equivalent to leaving the light gray shaded region (without exceeding $R_{0}=1$, the dashed line).

**Figure 5:**
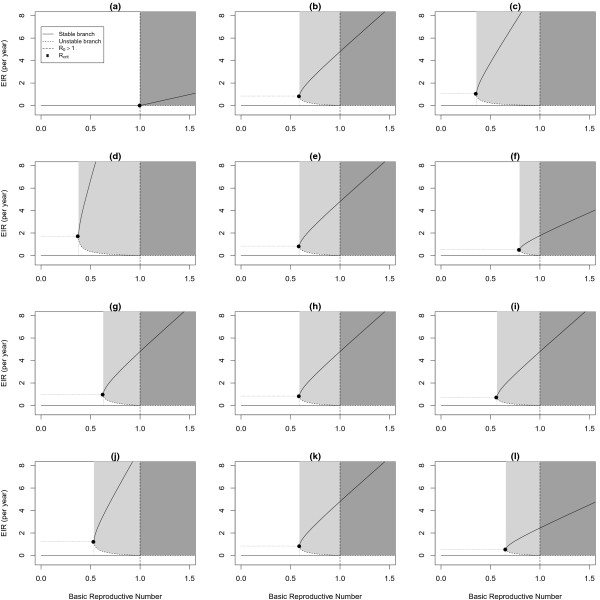
**Bifurcation diagrams changing model parameters individually (*ρ*,*σ*,*π*, and*γ***_***C***_**) while keeping all other parameters constant at the base model values (Figure**4**b).** The vertical dashed line shows $R_{0}=1$; the solid curve represents the stable equilibrium and the dotted curve shows the unstable equilibrium; the point shows $\left(R_{\text{crit}},\Lambda_{\text{crit}}\right)$; the light grey area shows the region of bistability– the area where malaria will persist, if endemic, but cannot invade; and the dark grey region shows where malaria can always invade and persist. In all of the bifurcation diagrams, $R_{0}$ ranged from 0 to 1.5 and $R_{\text{CC}}$ varied accordingly, changing only transmission rates (*τ*_*N*_ and *τ*_*C*_). **(a)**-**(c)** vary *ρ***(a)** The ratio $R_{\text{CC}}:R_{\text{NN}}$ is 1:2**(b)** The ratio $R_{\text{CC}}:R_{\text{NN}}$ is 2:1**(c)** The ratio $R_{\text{CC}}:R_{\text{NN}}$ is 4:1. **(d)**-**(f)** vary *σ***(d)***σ*=0.5, **(e)***σ*=0.7. **(f)***σ*=1. **(g)**-**(i)** vary *π* while keeping *γ*_*NC*_ constant **(g)***π*=0.66**(h)***π*=0.5**(i)***π*=0.33**(j)**-**(l)** vary *γ*_*C*_**(j)** clinically immune infections clear in 100 days **(k)** clinically immune infections clear in 200 days **(l)** clinically immune infections clear in 400 days.

We explored how different model parameters affected the bifurcation diagram (Figure 5), by varying each parameter individually. To vary $R_{0}$, we changed *τ*_*N*_ and *τ*_*C*_, while keeping their ratio constant. To vary *ρ* or to adjust *ρ* when necessary (e.g., when changing *σ*), we varied the ratio *τ*_*N*_:*τ*_*C*_.

We show that the region of bistability depends strongly on the ratio *ρ*. When *ρ* is large, bistability can occur even for large incidence rates, as shown in Figure 5. Consequently, if malaria were to be eliminated then re-introduced, it would jump to being endemic at a higher level in the population than when *ρ* is small. When *ρ* is large, *Λ*_*crit*_ (the value of *Λ* that corresponds to $R_{\text{crit}}$) is also large. A large value of *Λ*_*crit*_ means that the force of infection needs to be reduced by less to move below the unstable endemic equilibrium and be eliminated.

We also found that even when *ρ* is held constant, the individual components of *ρ* (*σ*, *π* and *γ*_*C*_) still affect the bifurcation diagram, as shown in Figure 5. When we increased *σ* (Figure 5) while holding *ρ* constant, the region of bistabililty decreased. When *σ* is small (Figure 5) the proportion of infections that occur in clinically immune people increases, thus increasing the accumulation of clinical immunity and magnifying its effect on transmission.

We also explored the dynamics when varying the recovery rates (Figure 5), while holding *ρ* constant. When individuals recover quickly, the area of bistability is large, since there are more susceptible clinically immune individuals in the population. When *γ*_*C*_ is large, *Λ*_*crit*_ is also large; this means that the force of infection needs to be reduced by less to drop below the unstable endemic equilibrium for malaria to be eliminated. Also, when *γ*_*C*_ is large, the value of *Λ* when $R_{0}=1$ is larger than when individuals recover slowly. So if malaria is eliminated then re-invades, it would jump to a high endemic level in the population. A study of malaria resurgence found many cases where incidence jumped to a high endemic level from low (or undetectable) levels [43]. We also change the proportion of naïve infections, *π*. When *π* is large, so is $R_{\text{crit}}$ and *Λ*_*crit*_ and as *π* is decreased, so is $R_{\text{crit}}$ and *Λ*_*crit*_, making the region of bistability larger.

## Discussion

The question of whether there are places where malaria is endemic, but where it could remain stably eliminated *under current transmission and treatment conditions* (ie., places where malaria is bistable) is important to interpreting malaria data and planning control measures. It has been suggested that treatment-seeking behavior can lead to bistability in the dynamics of malaria infections of humans [12].

In areas where malaria is bistable, an aggressive program that held malaria infection at low levels until clinical immunity wanes could result in the disease remaining absent even after the program is terminated. Malaria would not re-invade in this case because treatment seeking by non-immune infected individuals would lead to shorter duration of infectiousness and less overall transmission. This is a potentially risky strategy, however, because if malaria does re-invade such an area, the fact that clinical immunity has waned could lead to increased morbidity [9-11]. Mass drug administration (MDA) is a possible example of such an aggressive approach. Although MDA has so far proved unsuccessful in permanently interrupting malaria transmission, it is successful at reducing parasitemia and does temporarily reduce transmission [44]. Further investigation of factors underlying bistability could improve understanding of when and where MDA would be likely to lead to long-term elimination.

We analyzed a simple model, and found a simple criterion that determines whether bistability can occur. In particular, we found that the key quantity is the life-cycle “transmission effectiveness” of clinically immune individuals, relative to non-immune individuals. We encapsulate this relative infectiousness in a ratio, which we call *ρ* and show that bistability can occur when *ρ* exceeds 1+*D*/(*πL*), where *D* is the duration of naïve infection, *π* is the proportion of naïve cases that recover to become clinically immune, and *L* is the length of immunity. We also show that, in addition to duration of infection and ability to transmit the disease, the relative *susceptibility* of clinically immune individuals to new infections is a key component of this ratio. Although the relative susceptibility is a key component to understanding bistability in malaria, little is known about the relative susceptibility of clinically immune individuals.

Chiyaka et al. [14] discuss the importance of reducing the reproductive number under control efforts ($R_{C}$) below one to eliminate malaria and to gauge the size of an outbreak arising from an imported malaria case. They also point out the importance of investigating the stability of malaria elimination. Bistability of malaria, as we explore here, is one possible mechanism that could make malaria elimination more stable. Estimates of our criterion, *ρ*∗ could be a valuable component of efforts to assess the stability of malaria control in specific areas.

The details of clinical immunity to malaria are more complex than those in our simple model: in particular, clinical immunity continues to develop, there is not simply one kind of clinical immunity. Nonetheless, we expect our qualitative results to apply to more realistic situations. We expect the possibility of bistability when the relative life-cycle transmission effectiveness of clinically immune individuals is high. Thus, measuring the components of transmission effectiveness, both in clinically immune and non-immune individuals, is important for evaluating and planning malaria control efforts. Although certain aspects of malaria are well studied, it is surprisingly difficult to find information bearing directly on the components of transmission effectiveness, particularly in clinically immune individuals. Continued investigation of how these components determine transmission effectiveness will be important in understanding the population-level patterns of the spread and persistence of malaria.

Although duration of symptomatic infection is well studied [15,19], little is known about duration of asymptomatic infection [23]. This is a complicated question because malaria infections can be long and variable; failure to detect parasites may not mean an infectious event is over; conversely, parasites that are detected may be due to a new infectious event.

Transmission of infection to mosquitoes is another aspect of malaria biology that is not well understood. Although they are well studied individually, it is not clear how the components of transmission come together to affect the infectiousness per transmission event. These components include: gametocytemia [28,29]; TBI [30,33,34], which increases with gametocytemia, and wanes with age and clinical immunity; and other human and mosquito-specific factors [39]. More information on how these components interact to affect transmission would help to unravel how clinical immunity affects population-level transmission.

As we’ve shown, susceptibility of clinically immune individuals to malaria is an important component of the ratio of life-cycle transmission effectiveness. Susceptibility to new infection is known to be complex [38] but not well understood; in the literature “susceptibility” is frequently used to refer to susceptibility to clinical disease [38,39].

Our model neglects age structure, and oversimplifies the process of clinical immunity. In particular, the population is divided simply into those who are and are not clinically immune. Other omissions include seasonality, biting heterogeneity, and in fact, mosquitoes. For these reasons, the model is not expected to provide quantitatively accurate estimates of malaria dynamics.

The advantage of this simplistic approach, in our opinion, is that the model sheds light on the possible mechanisms and key quantities underlying bistability in malaria dynamics. In particular, we expect the importance of the quantity *ρ* – the ratio of life-cycle effectiveness of transmission between immune and non-immune individuals – to be robust to including more model details. Similarly, we expect some analogue of the time scale ratio *D*/(*πL*) – that is, the ratio between the time scales of infection and immunity – to be important in a detailed model.

## Conclusions

It has been suggested that human malaria-transmission dynamics exhibit bistability, which would have important implications for control efforts. We have shown that bistability through treatment seeking by clinically immune individuals is plausible in human malaria transmission dynamics. Using a simple model, we have demonstrated how these dynamics might play out, and determined key parameters underlying when and whether bistability might occur in real populations.

We find that the key quantities underlying whether bistability is expected to occur are: the relative “effectiveness” of clinically immune individuals, compared to naïve individuals, at transmission; and the time scale at which clinical immunity is lost, compared to the time scale of infectiousness. The model also shows that relative *susceptibility* to malaria infection should be considered part of transmission effectiveness, when making this comparison. We find that bistability can occur for plausible parameters, and suggest that more research into these two ratios may shed light on malaria dynamics, and guide future control efforts.

## Abbreviations

TBI: Transmission-blocking immunity; MDA: Mass drug administration.

## Competing interests

Both authors declare that they have no competing interests.

## Author’s contributions

LK and JD made and analyzed the model and drafted the manuscript. Both authors have read and approved the manuscript.

## Pre-publication history

The pre-publication history for this paper can be accessed here:

http://www.biomedcentral.com/1471-2334/13/428/prepub

## Supplementary Material

Additional file 1 Backwards bifurcation. Click here for file
