# Supplementary material for: Population-level effects of clinical immunity to malaria
Source: BMC Infect Dis. 2013 Sep 11;13:428. doi: 10.1186/1471-2334-13-428 (PMC3848694; doi:10.1186/1471-2334-13-428)
Supplement: Additional file 1 — Backwards bifurcation. [file 1471-2334-13-428-S1.pdf]

Additional File 1 – Backwards Bifurcation

To determine if bistability can occur, we analyze our model at the bifurcation point, $\mathcal{R}_0 = 1$, to determine the size of the ratio $\rho = \frac{\mathcal{R}_{NN}}{\mathcal{R}_{CC}}$ must be in order for a backwards bifurcation to occur.

To analyze our model, we can disregard $\frac{dS_N}{dt}$ because the population remains constant. So we re-write $\frac{dI_N}{dt}$, $\frac{dS_C}{dt}$, and $\frac{dI_C}{dt}$ in terms of I_N, S_C, I_C and T. We define Y to be the vector (I_N, S_C, I_C). We take the Jacobian of Y, $H(Y)$.

We then find the eigenvectors of the matrix $H(Y)$ at equilibrium (when Y=0. The eigenvectors determine whether or not a backwards bifurcation will occur at $R_0 = 1$. This is determined by the sign of the dominant eigenvectors of the Jacobian matrix [41]. The dominant right eigenvector, which gives the direction of the initial spread of the disease, is $V = [1, \frac{\gamma_{NC}}{\tau_N - \gamma_{NN} - \gamma_{NC} + \alpha}, 0]$ and the dominant left eigenvector which gives the contribution of each infected group to the overall spread is given by $W = [1, 0, \frac{\tau_C}{\tau_N - \gamma_{NN} - \gamma_{NC} + \gamma_C}]$.

To determine the criterion ρ^*, which gives the amount $\mathcal{R}_{CC}$ needs to be larger than $\mathcal{R}_{NN}$ in order for a backward bifurcation to occur, we look at the Jacobian matrix, H perturbed just a little from 0. Dushoff [41] showed that a backward bifurcation will occur if and only if

$$\rho* = W \cdot H_\varepsilon(0)V > 0 \tag{1}$$

For our model, we calculated ρ^* to be:

$$\rho* = 1 + \frac{\alpha + \mu}{\gamma_{NC}} \tag{2}$$

We take $\frac{1}{\alpha+\mu}$ to be the length of immunity (L), $\frac{1}{\gamma_{NN}+\gamma_{NC}}$ to be the duration of infection (D), and $\frac{\gamma_{NC}}{\gamma_{NN}+\gamma_{NC}}$. We find that when $\mathcal{R}_0 = 1$, bistability will occur when:

$$\rho* = 1 + \frac{D}{\pi L} \tag{3}$$
